# EBV-Induced LINC00944: A Driver of Oral Cancer Progression and Influencer of Macrophage Differentiation

**DOI:** 10.3390/cancers17030491

**Published:** 2025-02-01

**Authors:** Sawarot Srisathaporn, Tipaya Ekalaksananan, Chukkris Heawchaiyaphum, Sirinart Aromseree, David G. Maranon, Noelia H. Altina, Thawaree Nukpook, Jeffrey Wilusz, Chamsai Pientong

**Affiliations:** 1 Department of Microbiology, Faculty of Medicine, Khon Kaen University, Khon Kaen 40002, Thailand; sawarot.s@kkumail.com (S.S.); tipeka@kku.ac.th (T.E.); chukhe@kku.ac.th (C.H.); sirinar@kku.ac.th (S.A.); thawaree.n@kkumail.com (T.N.); 2 Department of Microbiology, Immunology, and Pathology, Colorado State University, Fort Collins, CO 80523, USA; david.maranon@colostate.edu (D.G.M.); noelia.altina@colostate.edu (N.H.A.); 3 HPV & EBV and Carcinogenesis Research Group, Khon Kaen University, Khon Kaen 40002, Thailand

**Keywords:** long non-coding RNA, oral squamous cell carcinoma, head and neck squamous cell carcinoma, Epstein–Barr virus, *LINC00944*, macrophage

## Abstract

Oral squamous cell carcinoma (OSCC) is a significant global health concern. Epstein–Barr virus (EBV) infection as well as long non-coding RNA (lncRNAs) associated EBV infection, have been linked to OSCC development and are known to influence cancer progression. *LINC00944* is associated with various cancers and immune cells, but its role in oral cancer remains underexplored. This study investigated the role of EBV-induced *LINC00944* in OSCC and its impact on the tumor microenvironment. The *LINC00944* expression was analyzed from a database of head and neck squamous cell carcinoma (HNSCC) tissues, and its expression in EBV-positive and EBV-negative OSCC cell lines was examined via qRT-PCR. We overexpressed *LINC00944* in SCC25 and ORL-48T oral cancer cell lines and evaluated its impact on migration and invasion ability using wound healing and transwell experiments. Additionally, we studied its influence on macrophage differentiation. The results showed that *LINC00944* expression was higher in HNSCC than in normal tissues and was linked to EBV-positive OSCC cell lines. *LINC00944* overexpressed-OSCC cell lines significantly increased cellular motility and invasiveness. Additionally, *LINC00944* was secreted in a cultured medium, delivered to macrophages, and promoted macrophage differentiation into the M1 subtype. Predicted interactions suggested that *LINC00944* targets miRNAs that regulate *NFKB1* and *RELA*. In conclusion, EBV-induced *LINC00944* contributes to OSCC progression by enhancing tumor cell migration, invasion, and macrophage differentiation, potentially regulating these processes through *NFKB1* and *RELA*. These findings provide valuable directions for *LINC00944*’s future studies on its mechanisms and suggest that it could be a target of study in EBV-associated OSCC.

## Simple Summary

This study investigated the role of long non-coding RNA *LINC00944* in oral squamous cell carcinoma (OSCC) and its association with cancer progression. Key findings indicated that *LINC00944* was upregulated in head and neck squamous cell carcinoma (HNSCC) tissues and Epstein–Barr virus (EBV)-positive oral cancer cell lines, suggesting its involvement in carcinogenesis and a potential influence of EBV infection on its gene expression. *LINC00944* promoted oral cancer cell progression by enhancing migration, invasion, and metastasis. Additionally, increased *LINC00944* expression corresponded with M1 macrophage polarization. The analysis of lncRNA-miRNA-mRNA interactions indicated that *LINC00944* might regulate *NFKB1* and *RELA* by targeting their miRNAs. EBV infection in epithelial cells altered the expression of *LINC00944*, which activated the TNFα/NF-κB signaling pathway. This activation boosted the expression of *NFKB1* and *RELA* through *LINC00944* regulation, likely involving microRNA interactions. This process promoted the expression of pro-inflammatory genes linked to cancer progression.

## 1. Introduction

Oral squamous cell carcinoma (OSCC) is a significant global health concern, accounts for over 90% of the cancer of the oral cavity, and is associated with high rates of morbidity and mortality [1]. The rising prevalence of oral cancer is exacerbated by factors such as tobacco, alcohol, betel quid, and oncogenic viruses [2,3,4]. Despite treatment advances, the 5-year survival rate for OSCC remains unimproved, and the rising prevalence of oral cancer is being worsened by diagnosis delays. Therefore, it is imperative that we enhance our understanding of OSCC cancer progression and identify potential therapeutic targets.

A common human gamma-herpesvirus known as Epstein–Barr virus (EBV) is implicated in several malignancies. Its biphasic life cycle is essential for its persistence and dissemination in human hosts, comprising latent and lytic phases. B lymphocytes predominantly harbor latent infections, whereas epithelial cells serve as sites for viral replication and amplification [5]. This virus is associated with oral lymphoproliferative diseases, including lymphoma of diffuse large B-cell, Burkitt’s lymphoma, and post-transplant lymphoproliferative disorder. As the EBV primarily transmits and persists in the oral cavity, it is also linked to oral carcinomas, such as OSCC, salivary gland epithelioma, and oral hairy leukoplakia [6]. EBV’s involvement in these malignancies varies, functioning either as a cofactor or contributing to tumor advancement by imparting malignant characteristics. The presence of EBV in OSCC varies among populations, with the range varying from 0 to 100% [7]. Several EBV oncoproteins and RNAs, including EBNAs, LMPs, and EBERs, have been associated with OSCC by promoting p53 inactivation [8] and EGFR transcription, inhibiting the apoptosis of cancer cells [8,9,10,11]. Moreover, EBV has been shown to induce epithelial-to-mesenchymal transition (EMT), enhance cell migration and invasion, and suppress apoptosis in OSCC cell lines in possible association with the IL6/JAK/STAT3 and TNF-α/NF-κB signaling pathways [12,13]. However, a previous study suggested that genetic alterations might also be required in EBV-associated cancers, which leads to ongoing debate about the association between oral cancer and EBV [14]. Therefore, additional studies on EBV’s involvement in the development of OSCC are evidently necessary.

Long non-coding RNAs (lncRNAs) are RNA molecules exceeding 200 nucleotides in length that do not contain open reading frames that are generally translated into proteins. LncRNAs have the potential ability to bind to DNA, RNA, and proteins, thereby enabling their participation in diverse cellular processes beyond gene expression regulation. As their roles in cellular functions, lncRNAs can be involved in chromatin modification and remodeling, mediate transcriptional activation or repression, guide proteins to specific areas within the genome, interact with mRNAs to modulate their durability, splicing, and protein synthesis, and serve as decoys for miRNAs [15].

In oral cancer, irregular lncRNA expression has been observed and is thought to contribute to disease progression [16,17]. Research has shown that viral proteins can influence lncRNA alterations, which are linked to the initiation and advancement of cancer [15]. Specifically, EBV infection has been found to modify lncRNA expression in infected cells and its associated cancers [18,19,20]. For example, upregulation of *APAF1-AS1*, *AL359062*, and *MALAT1* in nasopharyngeal carcinoma (NPC) [21] and *SNHG8*, *RP5-1039 K5.19*, and *TP73-AS1* in EBV associated-gastric cancer [22,23] can be associated with cancer promotion. Therefore, further study into the association of EBV-induced lncRNAs may help us understand how EBV infection is involved in carcinogenesis.

*LINC00944* has been associated with multiple cancers. Several studies have investigated its role in carcinogenesis and its involvement with cancer-related immune cells. An immune-related lncRNA study identified *LINC00944*, lncRNA associated with cancer, exhibits notable changes in expression across renal cell carcinoma (RCC), breast cancer, lung cancer, and colorectal malignancies. Its expression is modulated by the signaling pathway of the T-cell receptor [24]. In RCC, elevated *LINC00944* levels correlate with advanced tumor stages and poorer prognosis [25]. Moreover, *LINC00944* enhances proliferation, migration, and suppresses Akt phosphorylation [26]. In breast cancer, *LINC00944* expression is linked to *ADAR1* levels and immune signaling pathways, including tumor-infiltrating T lymphocytes. Lower *LINC00944* levels correlate with larger tumors, negative hormone receptor status, and poorer survival [27]. Elevated *LINC00944* expression is also associated with gastric cancer and lung cancer, such as lung squamous cell carcinoma and lung adenocarcinoma, and is used in prognostic risk score assessments and survival prediction models [28,29,30,31]. These studies provide insights into the tumor immune microenvironment and potential biomarkers for immunotherapy. However, *LINC00944*’s association with and impact on the carcinogenesis and tumor microenvironment in oral cancer remains unexplored.

The initiation, progression, and metastasis of OSCC are significantly influenced by the tumor microenvironment (TME). It is comprised of tumor cells, immune cells, endothelial cells, cancer-associated fibroblasts (CAFs), and the extracellular matrix (ECM), which collectively support tumor survival and progression. The interplay between tumor cells and TME components enables immune evasion, angiogenesis, and ECM remodeling, leading to treatment resistance [32]. Tumor-associated macrophages (TAMs) are pivotal in tumor progression and can polarize into pro-inflammatory M1 and immunosuppressive M2 phenotypes [33]. Recently, the link between TAMs and OSCC progression has also been investigated [34,35]. TAMs often undergo a shift toward an M2 phenotype, promoting tumor growth [36]. While M1 macrophages typically exhibit anti-tumor properties, some studies suggest they may also support tumor growth [37,38,39,40,41]. Additionally, in EBV-related tumors, EBV-carrying TAMs contribute to chronic inflammation [42] and can create an immunosuppressive tumor microenvironment, further aiding OSCC progression by hindering anti-tumor T-cell responses [43,44]. Consequently, investigating the role of *LINC00944* in the tumor microenvironment of oral cancer would be a valuable area of research.

In this study, we hypothesized that EBV might regulate the cellular lncRNA *LINC00944*, thereby facilitating the progression of oral cancer through direct effects on epithelial cancer cells or indirectly by modulating immune cells to create an environment conducive to cancer progression. Therefore, this study aimed to investigate the role of *LINC00944* in oral carcinogenesis and its impact on the tumor microenvironment. The data obtained demonstrated that *LINC00944* is significantly up-regulated in EBV-associated OSCC, suggesting EBV influences its expression. Overexpression of *LINC00944* in OSCC cell lines enhanced cancer cell migration and invasion. Secreted *LINC00944* was shown to affect neighboring cells and macrophage polarization, promoting M1 phenotypes. Through informatics interaction prediction, EBV-induced *LINC00944* may regulate oral cancer progression via the lncRNA-miRNA-mRNA axis. The study concludes that *LINC00944* may play an essential role in oral cancer metastasis and progression and could be suggested as a potential therapeutic target for oral cancer.

## 2. Materials and Methods

### 2.1. Expression Levels of LINC00944 in HNSCC from ImmReg Database

To evaluate *LINC00944* expression in HNSCC samples compared to normal controls, the dataset from ImmReg [24,45] was utilized to examine *LINC00944* in HNSCC cases. The analysis was conducted by ImmReg, which also generated the bar graph.

### 2.2. Cell Lines

The human tongue squamous cell carcinoma cell line, SCC25, was provided by Prof. Hironori Yoshiyama of Shimane University, Japan. The EBV-positive cell line, SCC25-EBV, was previously established by our colleagues [12]. The ORL-48T cell line, derived from human gingival squamous cell carcinoma, was supplied by Prof. Sok Ching Cheong from the Cancer Research Initiatives Foundation at Sime Darby Medical Centre Jaya, Malaysia. Furthermore, the human leukemia monocytic cell line, THP-1, was kindly provided by Prof. Greg Dean of Colorado State University, USA. All cell lines were cultured in Dulbecco’s Modified Eagle Medium/F12 (DMEM/F12) (Cat# 12500062, Gibco, Grand Island, NY, USA) except THP-1 cells, which were cultured in Roswell Park Memorial Institute (RPMI) 1640 (Cat# 31800022, Gibco, Grand Island, NY, USA). Each culture medium was supplemented with 10% fetal bovine serum (FBS) (Cat#6140079, Gibco, Grand Island, NY, USA) and 1% penicillin-streptomycin (Cat#15140122, Gibco, Grand Island, NY, USA). The cultures were maintained at 37 °C in a humidity-controlled incubator with a 5% CO_2_ atmosphere.

### 2.3. Quantitative Reverse Transcription PCR (qRT-PCR) for LINC00944 and Macrophage Markers Detection

The cell lines were extracted for the total RNA by using a TRIzol reagent (Cat#15596018, Invitrogen, Carlsbad, CA, USA). A RevertAid First Strand cDNA Synthesis Kit (Cat#K1622, Invitrogen, Carlsbad, CA, USA) and random hexamers (Cat#N8080127, Invitrogen, Carlsbad, CA, USA) were used to generate cDNA from 1 µg of RNA. The SsoAdvancedTM SYBR^®^ Green Supermix (Cat# 725271, Bio-Rad, Hercules, CA, USA) was used in the qRT-PCR reaction mixture and used an Applied Biosystems QuantStudio 6 Flex Real-Time PCR System (Applied Biosystems, Foster City, CA, USA) for the detection system. As an internal standard, the enzyme Glyceraldehyde 3-phosphate dehydrogenase *(GAPDH)* was employed. The 2^−ΔΔCT^ method [46] was used to determine the comparative mRNA expression levels. The Primer-BLAST tool was utilized for designing the primers used in this research [47]. The primer sequences are shown in Table 1.

### 2.4. Gene Over-Expression

The full length of *LINC00944* (NR_033878.1) was synthesized by IDT (Integrated DNA Technologies, Inc., Coralville, IA, USA) and cloned into the pEGFPN1 expression vector using T4 DNA ligase (Cat#M0202S, New England Biolabs, Ipswich, MA, USA). SCC25 and ORL-48T cells were either transfected with 2 μg of the pEGFPN1-*LINC00944* or pEGFPN1- control vector using jetprime^®^ transfection reagent (Cat#101000046, Polyplus-transfection SA, France) and after 48 h, the cells were used for further experiments.

### 2.5. Wound Healing Assay

A Wound Healing Assay was performed by plating cells in 24-well plates with specific densities: 3 × 10^5^ cells/well for SCC25-*LINC00944*, SCC25-pEGFPN1, ORL-48T-*LINC00944*, and ORL48T-pEGFPN1. The cells were cultured in DMEM/F12 medium containing 10% FBS and incubated at 37 °C in a 5% CO_2_ environment until reaching full confluence. A straight wound was then created using sterile 200 μL pipette tips, followed by gentle washing with 1X PBS twice. Subsequently, the cells were maintained in DMEM/F12 medium supplemented with 1% FBS. Wound closure was monitored at 0, 6, 12, 18, and 24 h for five areas per wound using a Nikon ECLIPSE-Ti2-U inverted fluorescence microscope and NIS-Elements Advanced Research Imaging Software version 3.0. The progression of wound closure was evaluated using ImageJ version 1.53k [48], with results expressed as a percentage relative to the initial wound width.

### 2.6. Transwell Assay

A Transwell insert with a 0.8 µm NEST^®^ polycarbonate membrane was coated with 50 μL of 1 mg/mL Matrigel Matrix (Corning, Bedford, MA, USA). The assay was conducted by seeding cells in the coated insert at specific densities: 3 × 10^5^ cells/well for SCC25-*LINC00944*, SCC25-pEGFPN1, ORL-48T-*LINC00944*, and ORL48T-pEGFPN. Cells were incubated for 24 h in a 5% CO_2_ environment. Subsequently, non-invasive cells on the upper surface were eliminated. Invasive cells that had penetrated the matrix gel and adhered to the lower surface underwent fixation using 70% ethanol. These cells were then stained with 0.2% crystal violet, imaged, and quantified across five fields. Image capture was conducted using a Nikon ECLIPSE-Ti2-U inverted fluorescence microscope (Nikon Corporation, Tokyo, Japan). The NIS-Elements Advanced Research Imaging Software version 3.0 (Nikon Corporation, Tokyo, Japan) was employed for image acquisition and analysis.

### 2.7. Association Between LINC00944 and Immune Cell Analysis Tools

To investigate the association between *LINC00944* and immune cells, we obtained analyzed data of *LINC00944* and immune cells in HNSCC cases HNSCC cases from ImmReg (accessed on 22 September 2024). These data included information on *LINC00944* and gene-associated immune cells, which had been processed through the ImmReg platform using various analytical tools, including XCELL [49], TIMER [50,51], QUANTISEQ [52], MCPCOUNT [53], EPIC [54], and CIBERSORT [55], to examine the connection between lncRNA and immune cells linked to specific genes. SRplot [56] was utilized to create the map, with each region’s size reflecting its significance within the group.

### 2.8. Conditioned Medium Preparation

SCC25 cells and ORL-48T cells were either transfected with pEGFPN1-*LINC00944* vector or pEGFPN1- control vector using jetprime^®^ transfection reagent (Cat#101000046, Polyplus-transfection SA, France) for 48 h. Subsequently, the transfected cells were rinsed twice with PBS. Fresh culture medium was then introduced, and the cells were incubated for an additional 48 h. The conditioned medium was collected and centrifuged at 500× *g* 10 min twice, followed by 2000× *g* 15 min twice. The collected condition medium was used as a medium for culturing another set of SCC25 cells, ORL-48T cells, and THP-1 cells for 48 h and the cells were collected for further experiments.

### 2.9. Macrophage Differentiation

THP-1 cells were used for the macrophage differentiation study. Firstly, THP-1 cells were activated into M0 by culturing in 10 ng/mL PMA (Cat#P8139, Sigma-Aldrich, St. Louis, MO, USA) in RPMI1640 supplemented 10% FBS for 48 h followed by washing the PMA off and a 48 h rest period in fresh media before exposure to polarizing cytokines. PMA-activated THP-1 cells were differentiated into M1 by treating the cells with 1 ng/mL of LPS (Cat# L4391, Sigma-Aldrich, St. Louis, MO, USA) and 20 ng/mL IFN-g (Cat #300-02, PeproTech, Cranbury, NJ, USA) for 48 h. For M2, PMA-activated THP-1 cells were treated with 20 ng/mL IL-4 (Cat#200-04, PeproTech, Cranbury, NJ, USA), 20 ng/mL IL-13 (Cat#200-13, PeproTech, Cranbury, NJ, USA), and 20 ng/mL IL-10 (Cat#200-10, PeproTech, Cranbury, NJ, USA), for 48 h before RNA harvesting, cDNA synthesis, and RT-PCR for M1 and M2 marker transcript amplification.

### 2.10. In Vitro Transcription

The 1 µg of linearized DNA template was mixed with 5× transcription buffer (Cat# 18033019, Thermo Fisher Scientific, Carlsbad, CA, USA), T7 RNA polymerase (Cat# 18033019, Thermo Fisher Scientific, Carlsbad, CA, USA), ribonucleotides (Cat# R0481, Thermo Fisher Scientific Baltics UAB, Vilnius, Lithuania), and Ribolock RNA inhibitor (Cat# EO0382, Thermo Fisher Scientific Baltics UAB, Vilnius, Lithuania). The reaction was incubated at 37 °C for 3 h in a thermocycler machine. Then, the mixture was combined with DNase I (Cat# EN0521, Thermo Fisher Scientific Baltics UAB, Vilnius, Lithuania) to remove the DNA template, mixed, and then incubated at 37 °C for 15 min. The RNA was subjected to purification through a phenol-chloroform extraction followed by ethanol precipitation. RNA was quantified using the NanodropTM 2000/2000c spectrophotometer (Thermo Scientific, USA).

### 2.11. Transfection of In Vitro Transcripts of LINC00944

THP-1 cells were induced to M0 macrophages by treatment with 10 ng/mL PMA (Sigma-Aldrich, St. Louis, MO, USA) in RPMI1640 supplemented with 10% FBS and 1% penicillin-streptomycin (Gibco, Grand Island, NY, USA) for 48 h, followed by gentle washing with 1X PBS twice and resting for 48 h in fresh media. The culture medium was removed and replaced with fresh media prior to transfection. In vitro transcripts of *LINC00944* were transfected into THP-1 cells using Lipofectamine MessengerMAX Transfection Reagent (ThermoFisher Scientific, Waltham, MA, USA). Briefly, a volume of 1.5 µL of transfection reagent was mixed in 25 µL of Opti-MEM (Gibco, Grand Island, NY, USA), vortexed for 2 sec, and incubated for 10 min at room temperature. One microgram of in vitro transcripts of *LINC00944* was diluted in 25 µL Opti-MEM medium and vortexed for 2 sec. The mixture of transfection reagent was transferred to the tube containing diluted lncRNA and gently mixed by pipetting up and down ten times, followed by incubation at room temperature for 5 min. Fifty microliters of the mixture containing transfection reagent and lncRNA were added to cells in a 24-well plate. The cells were incubated with the transfection mixture in a cell culture incubator at 37 °C in a humidified atmosphere with 5% CO_2_ for 48 h, after which the cells were collected for expression determination experiments.

### 2.12. Digital Droplet PCR (ddPCR) for LINC00944 and Macrophage Marker Detection

The mixture of ddPCR consisted of 10 μL QX200 ddPCR EvaGreen Supermix (Cat#186-4033, Bio-Rad, Hercules, CA, USA) and 4 nM of each primer in a total volume of 20 μL. This reaction was transferred to the sample well of a droplet cartridge (Cat#186-3008, Bio-Rad, Hercules, CA, USA), while 70 μL of QX200 Droplet Generation Oil for EvaGreen (Cat#186-4005, Bio-Rad, Hercules, CA, USA) was added to the oil well. Following Bio-Rad’s protocol, a gasket (Cat#186-3009, Bio-Rad, Hercules, CA, USA) was positioned on the cartridge before placing it in the droplet generator (Cat#186-3002 Bio-Rad, Hercules, CA, USA). The generator created approximately 20,000 individual droplets suspended in an emulsion. This emulsion was then moved to a 96-well plate (Cat#0030128591, Eppendorf, Hamburg, Germany) and sealed using a Pierceable foil heat seal (Cat#181-4040, Bio-Rad, Hercules, CA, USA) with a PX1 PCR Plate Sealer (Cat#181-4000, Bio-Rad, Hercules, CA, USA). The sealed plates underwent thermal cycling in a T100 Thermal Cycler (Cat#1861096, Bio-Rad, Hercules, CA, USA) following Bio-Rad’s standard EvaGreen protocol. Post-amplification, the plate was analyzed using a Bio-Rad droplet reader (Cat#1861096, Bio-Rad, Hercules, CA, USA), and raw fluorescence amplitude data were extracted from the Quantasoft software (Bio-Rad, Hercules, CA, USA) for further analysis. The primer sequences are shown in Table 1.

### 2.13. Microarray Data

Microarray data of SCC25 cells and EBV-positive SCC25 cells were previously analyzed by our colleagues [12]. The extraction of RNA was performed using an ISOGEN reagent (Nippon Gene, Tokyo, Japan), following the instructions provided by the manufacturer. To assess the RNA’s quantity and quality, researchers employed a Qubit 2.0 fluorometer (Invitrogen, Carlsbad, CA, USA) and a 2100 Bioanalyzer (Agilent, Santa Clara, CA, USA). The Agilent G4900DA SureScan Microarray Scanner System (Agilent, Santa Clara, CA, USA) was employed following the manufacturer’s specifications. DNA Chip Research Inc. (Tokyo, Japan) performed the microarray data analysis. Genes demonstrating a fold change greater than 1.5 were selected for further investigation (Supplementary Table S1), and a heatmap was generated utilizing GraphPad Prism version 10.1.1 (GraphPad Software Inc., San Diego, CA, USA). 

### 2.14. Target Prediction Tools

The interaction of the lncRNA *LINC00944* and miRNA was analyzed by Pre-computed Predictions: RNA22 versions 1.0 and 2.0 (https://cm.jefferson.edu/rna22/Precomputed/, accessed on 8 November 2024) [57], miRanda tool version 3.3a [58] via SRplot web base (https://www.bioinformatics.com.cn/en, accessed on 8 November 2024) [56], and IntaRNA version 5.0.10 (http://rna.informatik.uni-freiburg.de, accessed on 8 November 2024) [59,60,61,62]. The miRNA-related pathways were analyzed by miRPath version 4.0 (http://62.217.122.229:3838/app/miRPathv4, accessed on 12 November 2024) [63]. Several databases were utilized to investigate the prediction of miRNAs to target mRNAs. These included MiRDB (https://mirdb.org/, accessed on 13 November 2024) [64,65], which employed a predicted score threshold of ≥50; mirDIP Version 5.3.0.2, Database version 5.2.3.1 (https://ophid.utoronto.ca/mirDIP/, accessed on 13 November 2024) [66,67], using high to very high predicted scores; miRTarBase (https://mirtarbase.cuhk.edu.cn/, accessed on 13 November 2024) [68], requiring at least one binding site; and miRWalk version 3.0 (http://mirwalk.umm.uni-heidelberg.de, accessed on 13 November 2024) [69], also necessitating a minimum of one binding site.

### 2.15. Statistical Analysis

Data analysis was conducted using GraphPad Prism version 10.1.1 (GraphPad Software Inc., San Diego, CA, USA). The Shapiro–Wilk test was employed to assess the normality of data distribution. To determine differences between two independent groups, *t*-tests were utilized. For comparisons involving more than two independent groups, one-way ANOVA was applied. Each experiment was conducted in triplicate. A *p*-value less than 0.05 was considered statistically significant.

## 3. Results

### 3.1. LINC00944 Is Over-Expressed in Head and Neck Squamous Cell Carcinoma (HNSCC) Tissues and Is Associated with EBV Infection in Oral Cancer

**Figure 1:**
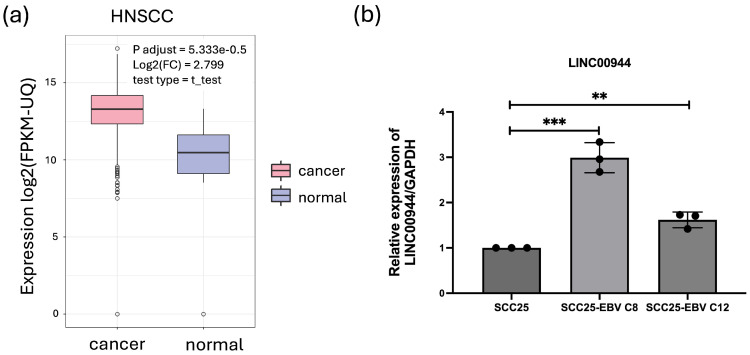
The expression level of *LINC00944* was significantly increased in HNSCC patients and EBV-positive OSCC cell lines. (**a**) *LINC00944* expression levels in HNSCC tumor and adjacent tissues from the ImmReg database. (**b**) Relative expression levels of *LINC00944* in EBV-positive SCC25 cells clone 8 (C8) and clone 12 (C12) compared with that EBV-negative and SCC25 cells, the data were analyzed using *t*-tests. (** *p* < 0.01; *** *p* < 0.001).

To explore the role of *LINC00944* in oral carcinogenesis, we first investigated the association of *LINC00944* expression level in HNSCC tissues and normal tissues using the analyzed data from ImmReg database. As expected, HNSCC tissues exhibited a significant upregulation of *LINC00944* expression when compared to normal tissue samples (Figure 1a). This alteration in *LINC00944* expression in cancer tissues could suggest its potential involvement in carcinogenesis. Furthermore, oral cancers are categorized as a subtype of HNSCC. Therefore, *LINC00944* might also contribute to the process of oral carcinogenesis.

Previously, our colleague conducted a microarray experiment to investigate gene expression patterns of the human tongue squamous cell carcinoma cell line (SCC25) and EBV-positive SCC25 cell line (SCC25-EBV) [12]. Analyzing this microarray dataset, we found that *LINC00944* was upregulated in SCC25-EBV cells when compared to SCC25 cells (log2FC = 3.67, *p*-value < 0.05). To validate this observation, the expression of *LINC00944* was determined in SCC25 and SCC25-EBV clone 8 (C8) and clone 12 (C12) cell lines using qRT-PCR. Consistent with the result from microarray data, *LINC00944* expression was significantly higher in EBV-positive SCC25 C8 and C12 cells when compared to EBV-negative SCC25 cells (Figure 1b). Thus, these findings suggest that EBV infection has an impact on the expression of *LINC00944*.

### 3.2. EBV-Induced LINC00944 Enhanced Oral Cancer Cell Migration and Invasion

**Figure 2:**
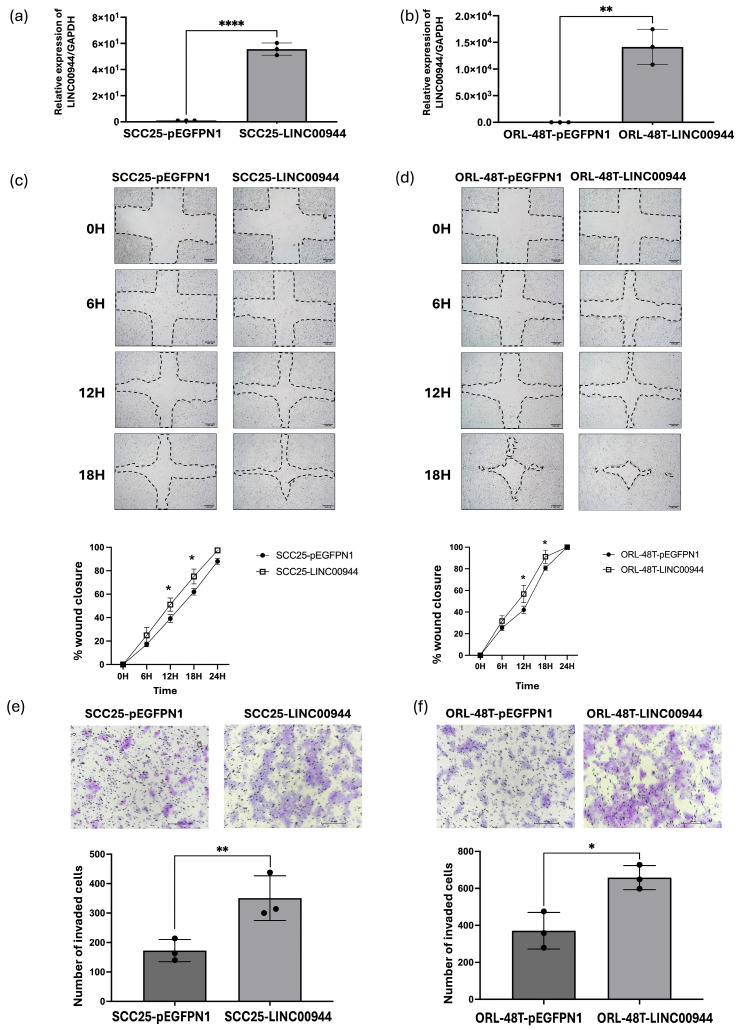
Biological consequence study. (**a**) *LINC00944* expression levels in SCC25 cells with *LINC00944* overexpression and (**b**) ORL-48T cells were quantified using qRT-PCR. (**c**) The impact of *LINC00944* on SCC25 cell migration and (**d**) ORL-48T cells overexpressing *LINC00944* was evaluated through a wound healing assay. Wound closure was measured at 0, 6, 12, 18, and 24 h and presented as a percentage of the initial wound width; scale bar: 100 μm. (**e**) The invasive potential of SCC25 cells and (**f**) ORL-48T cells overexpressing *LINC00944* was assessed via a Transwell assay, with invaded cells counted in five fields; Scale bar: 100 μm. Statistical analysis was performed using *t*-tests. (* *p* < 0.05; ** *p* < 0.01; **** *p* < 0.0001).

To investigate the biological impact of *LINC00944* on the development of oral cancer cells, we used SCC25 and ORL-48T cell lines to establish overexpressed *LINC00944* (SCC25-*LINC00944* and ORL-48T-*LINC00944*) and their respective control cells (SCC25-pEGFPN1 and ORL-48T-pEGFPN1). As expected, the expression of *LINC00944* was significantly increased in SCC25 and ORL-48T cells that had been transfected with a *LINC00944*-carrying expression plasmid when compared to the control cells (Figure 2a,b).

The study of cell migration and invasion in oral cancer cells was performed using SCC25-*LINC00944* and ORL-48T-*LINC00944* cells as *LINC00944* overexpressed cells while SCC25-pEGFPN1 and ORL-48T-pEGFPN1 as their counterpart control cells. The investigation of cell migration using a wound healing assay demonstrated that the size of wound closure in SCC25-*LINC00944* and ORL-48T-*LINC00944* was significantly decreased 12 h after the wound was created when compared to their counterpart control cells that did not over-express the lncRNA (Figure 2c,d). The finding suggests that *LINC00944* overexpression enhanced the motility of both SCC25 and ORL-48T cells. Furthermore, observation of cell invasion through a transwell assay revealed that elevated *LINC00944* expression in oral cancer cells increases invasive ability in both SCC25 and ORL-48T cell lines (Figure 2e,f). Therefore, these findings suggest that increased *LINC00944* levels enhanced the migratory and invasive capabilities of oral cancer cells, which consequently promote tumor progression.

### 3.3. Secreted EBV-Induced LINC00944 Was Delivered to Neighbor Cells

**Figure 3:**
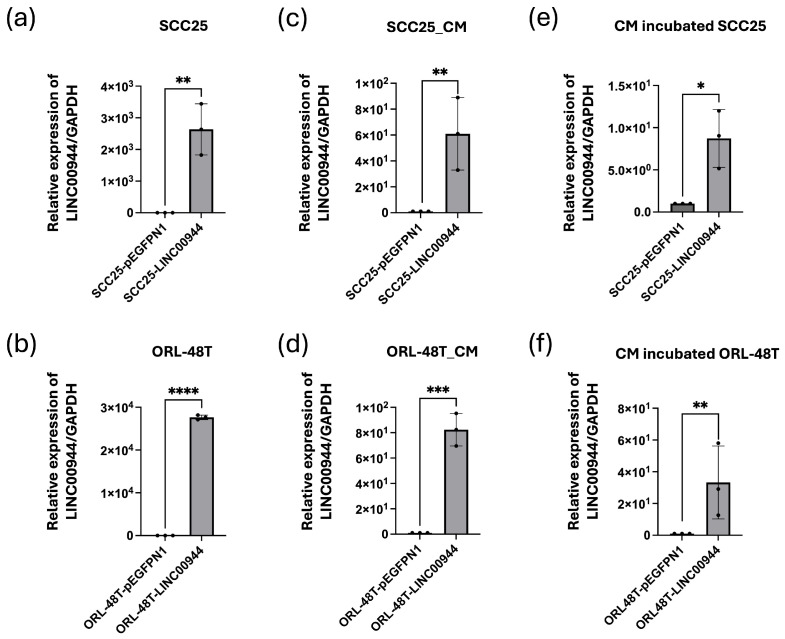
Secreted EBV-induced *LINC00944* can be delivered to neighbor cells. The expression level of *LINC00944* in cells was determined by qRT-PCR. (**a**) Relative expression of *LINC00944* in SCC25 cells overexpressing *LINC00944* and (**b**) ORL-48T cells four days after transfection. (**c**) Relative expression of *LINC00944* in conditioned medium (CM) collected from SCC25 cells overexpressing *LINC00944* and (**d**) ORL-48T cells cultured media. (**e**) Relative expression of *LINC00944* in SCC25 and (**f**) ORL-48T cells incubated with *LINC00944*-conditioned medium. The data were tested using *t*-tests. * *p* < 0.05; ** *p* < 0.01; *** *p* < 0.001; **** *p* < 0.0001).

To investigate whether *LINC00944* can be secreted from cells, overexpression of *LINC00944* in SCC25 and ORL-48T cells was induced by transfecting with the expression plasmids. After 48 h of transfection, the cell culture medium was replaced with fresh medium, and cells were incubated for an additional 48 h. The conditioned medium (CM) was then collected, and the expression level of *LINC00944* was measured by qRT-PCR. The results showed significantly higher levels of *LINC00944* in the overexpression plasmid-transfected group when compared to the empty plasmid-transfected group (Figure 3a,b). Similar results were observed in the cultured conditioned medium (Figure 3c,d), indicating that *LINC00944* can be secreted from the cells.

To further investigate whether secreted *LINC00944* in the conditioned medium can be delivered to and taken up by oral epithelial cancer cells, SCC25 and ORL-48T cells, were incubated with the conditioned medium from *LINC00944* overexpressing cells or control cells. The results showed that levels of *LINC00944* were significantly increased in the transfected group (Figure 3e,f), suggesting that secreted *LINC00944* can be delivered to and taken up by neighboring cells.

### 3.4. Secreted EBV-Induced LINC00944 Was Delivered to THP-1 Cells and Induced an M1 Phenotype

**Figure 4:**
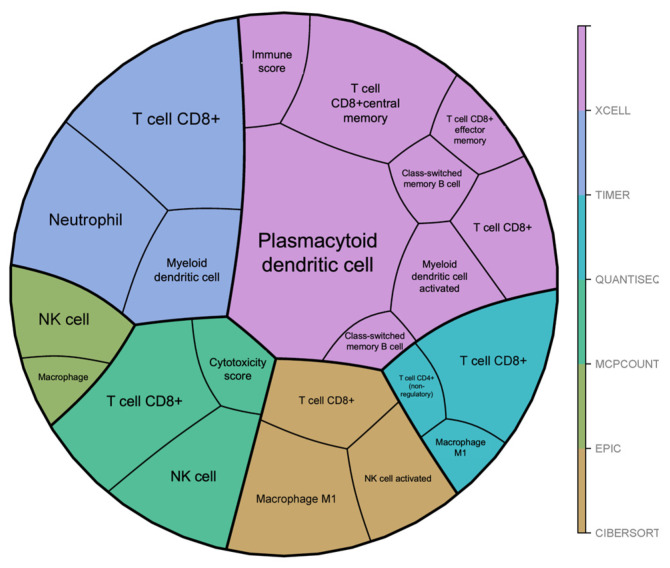
The illustration of *LINC00944* associated immune cell populations from gene expression datasets of HNSCC from ImmReg databases analyzed by multiple analysis tools including XCELL, TIMER, QUANTISEC, MCPCOUNT, EPIC, and CIBERSORT.

According to our findings in Figure 3c,d, *LINC00944* can be secreted from the cells; therefore, EBV-induced lncRNA may promote oral cancer progression by involving immune cells in the TME. To explore the association between *LINC00944* and immune cells, we analyzed gene expression data from HNSCC cases using various analysis tools, including XCELL, TIMER, QUANTISEQ, MCPCOUNT, EPIC, and CIBERSORT. The data showed the association between *LINC00944* and immune cells in different subtypes, including CD8 + T cells, CD4 + T cells, dendritic cells, NK cells, macrophages and M1 macrophages, neutrophils, and B-cells (Figure 4 and Table 2).

Based on our results in Figure 3, *LINC00944* was secreted from oral cancer cells, suggesting its potential role in influencing not only intracellular processes but also the surrounding environment. Consequently, we further aimed to explore the association between *LINC00944* and macrophages.

**Figure 5:**
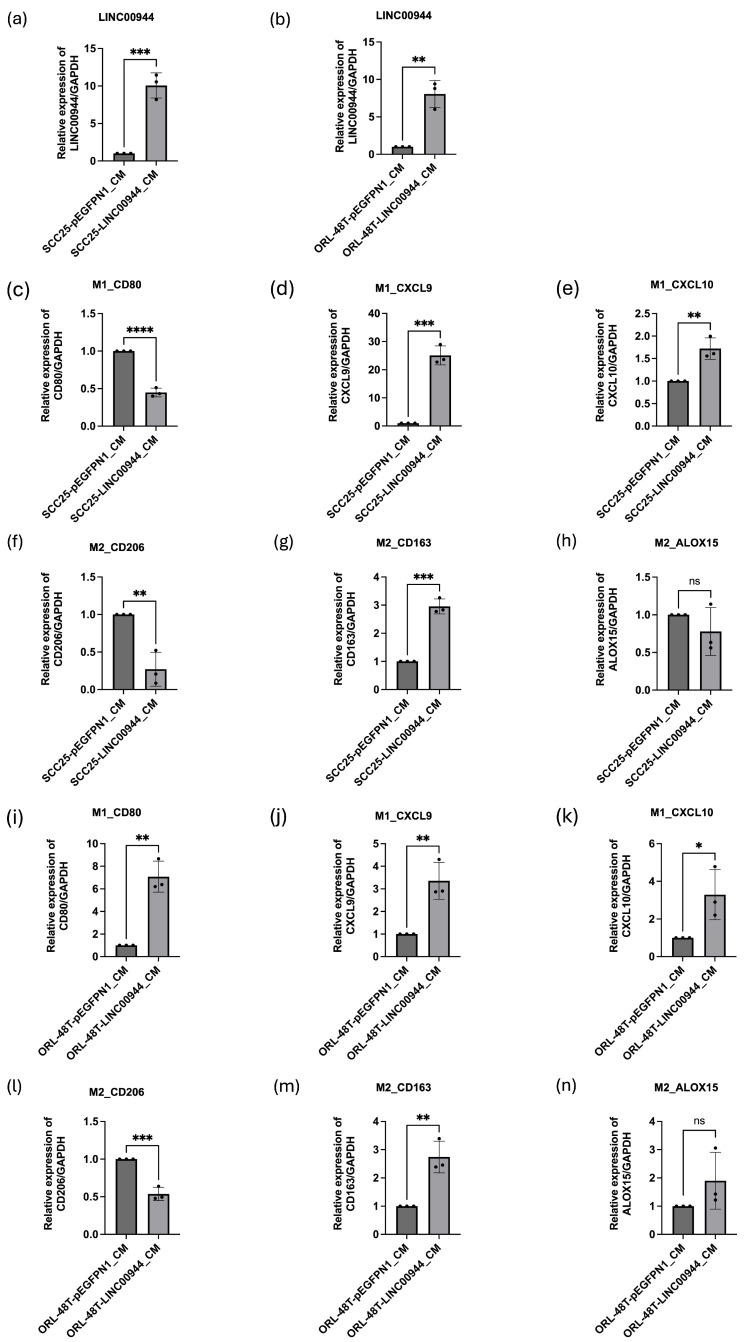
Secreted EBV-induced *LINC00944* was delivered to THP-1 cells and induced M1 phenotype. The expression level of *LINC00944* in cells was determined by qRT-PCR. (**a**) Relative expression of *LINC00944* in THP-1 cells, which incubated conditioned medium from SCC25 cells overexpressing *LINC00944* and (**b**) ORL-48T cells. Relative expression of M1 markers (**c**–**e**) and M2 markers (**f**–**h**) in THP-1 cells in conditioned medium collected from SCC25 cells overexpressing *LINC00944*. (**i**–**k**) M1 markers and (**l**–**n**) M2 markers in THP-1 cells in a conditioned medium collected from ORL-48T cells overexpressing *LINC00944*. M1 markers; *CD80*, *CXCL9*, *CXCL10*, and M2 markers; *CD206*, CD*163*, *ALOX15*. The data were tested using *t*-tests. (* *p* < 0.05; ** *p* < 0.01; *** *p* < 0.001; **** *p* < 0.001; ns non-significant).

To investigate whether THP-1 cells could internalize secreted *LINC00944*, we incubated THP-1 cells with a conditioned medium collected from *LINC00944*-expressing epithelial cells (SCC25-*LINC00944* and ORL-48T-*LINC00944* cells) as well as their counterpart control cells (SCC25-pEGFPN1 and ORL-48T-pEGFPN1 cells), then its expression level was determined by qRT-PCR. As expected, the expression levels of *LINC00944* were significantly up-regulated in THP-1 cells exposed to *LINC00944*-conditioned medium compared to the control group (Figure 5a,b). Therefore, this finding suggests that *LINC00944* uptake by THP-1 cells might influence monocyte behavior within the TME.

Additionally, we further assessed the impact of *LINC00944* on THP-1 cells by determining the expression level of macrophage subtype markers for M1 (*CD80*, *CXCL9*, and *CXCL10*) and M2 (*CD206*, *CD163*, and *ALOX15*) phenotypes using qRT-PCR. In THP-1 cells incubated with *LINC00944*-conditioned medium from SCC25 cells, M1 markers, *CXCL9*, and *CXCL10,* were significantly up-regulated, along with M2 marker, *CD163*, while M1 marker, *CD80*, and M2 marker, *CD206*, were significantly downregulated when compared to control cells (Figure 5c–h). Similarly, THP-1 cells incubated with *LINC00944*-conditioned medium from ORL-48T showed up-regulation of *CD80*, *CXCL9*, *CXCL10*, and *CD163*, with a concomitant down-regulation *CD206* (Figure 5i–n). Collectively, these results suggest that *LINC00944*, secreted by oral cancer cells, may promote an M1-like macrophage phenotype.

### 3.5. Overexpression of LINC00944 Directly Affects Monocyte Differentiation into the M1 Macrophage Subtype

**Figure 6:**
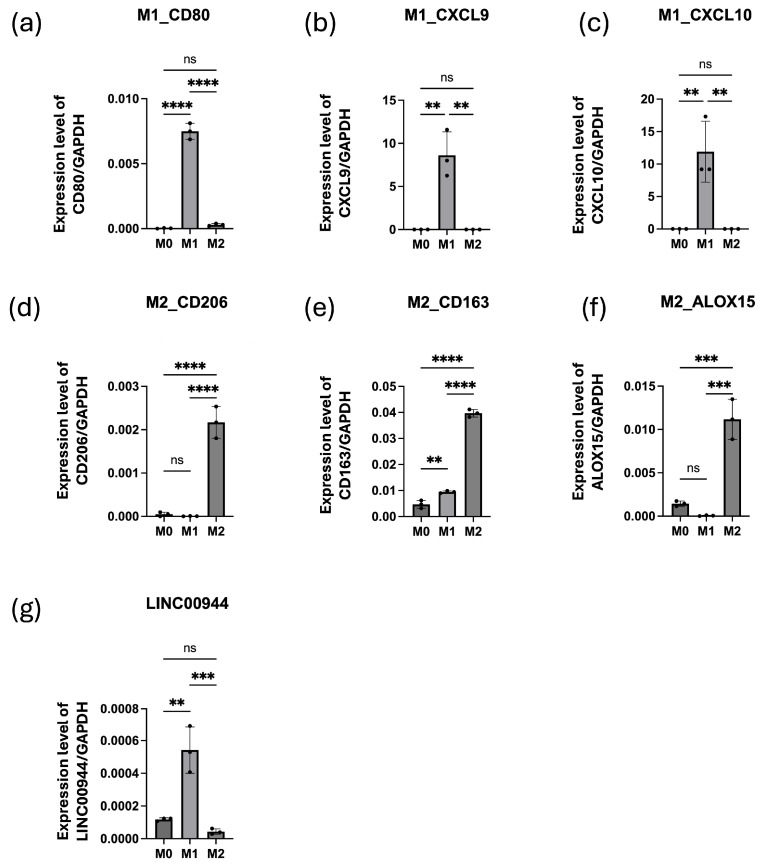
A high expression of *LINC00944* is observed in the M1 macrophage subtype. The expression level of M1 and M2 markers in M0, M1 (IFN-γ + LPS) and, M2(IL-4/IL-13/IL-10) cells was determined by ddPCR. M1 markers; (**a**) *CD80*, (**b**) *CXCL9*, and (**c**) *CXCL10*. M2 markers; (**d**) *CD206*, (**e**) CD*163*, and (**f**) *ALOX15*. (**g**) The expression level of *LINC00944* in M0, M1 (IFN-γ + LPS) and M2(IL-4/IL-13/IL-10) cells was determined by ddPCR. The data were tested using one-way ANOVA. (** *p* < 0.01; *** *p* < 0.001; **** *p* < 0.001; ns non-significant).

To investigate whether *LINC00944* is directly involved in M1/M2 differentiation, we first generated activated THP-1 cells into M0 (induced with PMA), followed by inducing polarization into M1 (induced by IFN-γ + LPS) and M2 (induced by IL-4/IL-13/IL-10) macrophages in vitro. For the detection of M1 and M2 macrophage, the expression of M1 and M2 markers was determined by ddPCR. As expected, M1-associated markers, *CD80*, *CXCL9*, and *CXCL10*, were significantly up-regulated in M1 (Figure 6a–c), while M2-associated markers, *CD163*, *CD206*, and *ALOX15*, were significantly up-regulated in M2 (Figure 6d–f). These results confirmed that distinct macrophage subtypes were developed by the polarization conditions used in this study.

Next, we investigated whether the alteration of *LINC00944* expression is associated with M1 and M2 polarization. The ddPCR analysis result showed that *LINC00944* expression was significantly up-regulated in M1 macrophages and down-regulated in M2 macrophages (Figure 6g). These findings suggest a positive association between *LINC00944* and M1 macrophage phenotype.

**Figure 7:**
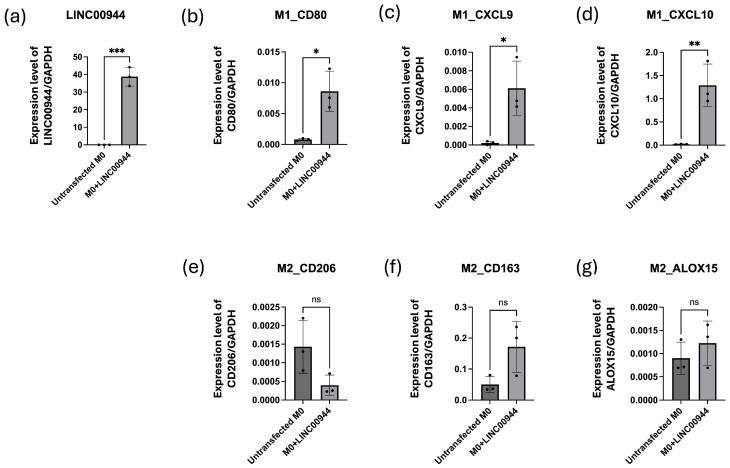
Overexpression of *LINC00944* directly affects M0 macrophage differentiation into the M1 macrophage subtype. (**a**) The expression level of *LINC00944* in transfected cells compared to untransfected cells. The expression level of M1 and M2 markers in untransfected THP-1 cells and *LINC00944* transfected THP-1 cells was determined by ddPCR. M1 markers; (**b**) *CD80*, (**c**) *CXCL9*, and (**d**) *CXCL10*. M2 markers; (**e**) *CD206*, (**f**) CD*163*, and (**g**) *ALOX15*. ddPCR was used to determine the expression level of *LINC00944*. The data were tested using *t*-test. (* *p* < 0.05; ** *p* < 0.01; *** *p* < 0.001; ns non-significant).

To study the effects of EBV-induced lncRNA *LINC00944* on macrophage differentiation, full-length *LINC00944* transcripts were transfected into PMA-activated THP-1 cells or M0 macrophages for 48 h. The expression of *LINC00944*, M1, and M2 macrophage markers was determined using ddPCR. The results showed that the expression level of *LINC00944* was significantly higher in cells transfected with *LINC00944* when compared to untransfected cells (Figure 7a). Overexpression of *LINC00944* significantly up-regulated the M1 markers, *CD80*, *CXCL9*, and *CXCL10* (Figure 7b–d), while showing no significant changes in the M2 markers, *CD206*, *CD163*, and *ALOX15* (Figure 7e–g). Therefore, these findings suggest that EBV-induced lncRNA *LINC00944* directly promotes the differentiation of macrophages into the M1 subtype.

### 3.6. LINC00944 Interaction with miRNA Which Specifically Targets NF-κB Signaling Pathway

**Figure 8:**
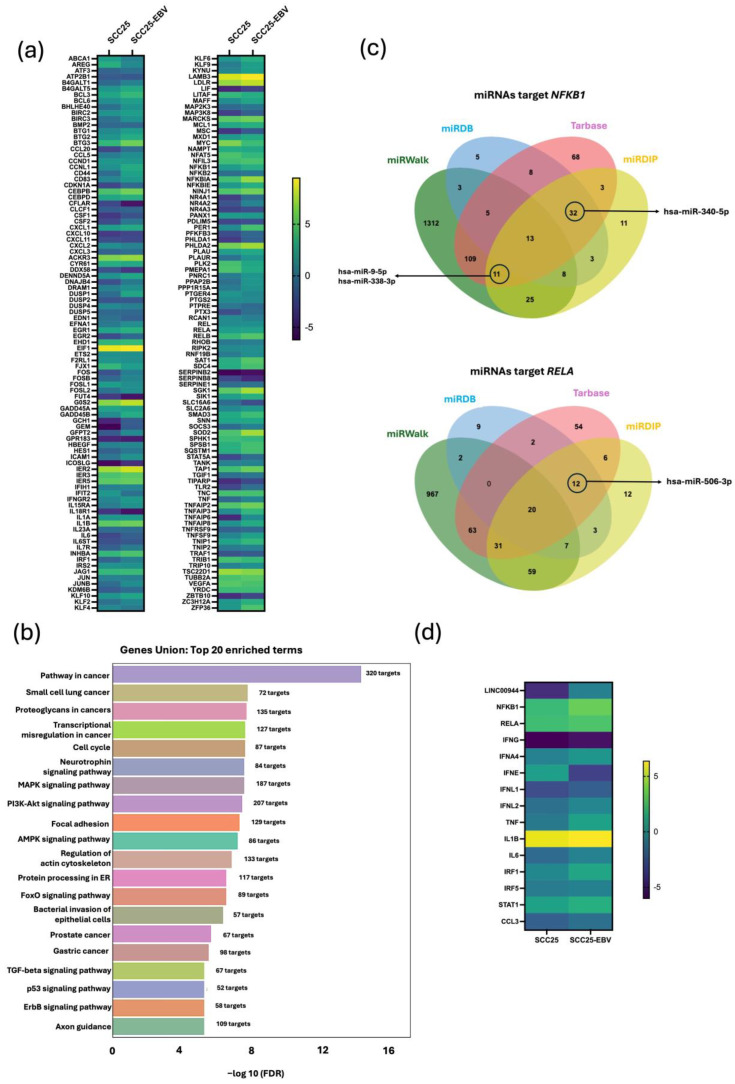
(**a**) The differential expression of genes (DEGs) of TNF-α/NF-κB signaling pathway from microarray data in EBV positive SCC25 compared to EBV negative SCC25 cell lines. (**b**) miRNA-associated pathways from the miRNAs predicted to be targeted by *LINC00944*. (**c**) Jvenn illustrated miRNAs that were identified as targets of *NFKB1* and *RELA* from four databases. The targeted miRNAs that are downregulated in oral cancer and predicted to be targeted by *LINC00944* were indicated. (**d**) DEGs of M1 macrophage polarization genes.

The TNF-α/NF-κB signaling pathway is recognized as a common hallmark in EBV-associated epithelial cancers [13] and is associated with OSCC migration and invasion [70]. Thus, the role of EBV-induced *LINC00944* in oral carcinogenesis was examined through the TNF-α/NF-κB signaling pathway. The differential gene expression related to the TNF-α/NF-κB signaling pathway, as analyzed from microarray data of SCC25-EBV, is shown in Figure 8a.

The function of lncRNA as competing endogenous RNA (ceRNA), one of the roles of lncRNA in cellular functions, was investigated through the identification of potential interactions between *LINC00944* and miRNAs. The prediction of target miRNAs that the lncRNA might sponge was performed using the RNA22 tool for selection and then the interactions were confirmed by miRanda and IntaRNA. Predicted miRNAs that have been reported to be downregulated in oral cancers were selected as candidate miRNAs. Among these, hsa-miR-26a/b [71], hsa-27a-3p [72], hsa-miR-125b [73], hsa-miR-1271 [74], hsa-miR-338 [75], hsa-miR-340 [76], hsa-miR-9 [77], and hsa-miR-506 [78] were predicted to be targeted by *LINC00944*. Notably, these miRNAs have been observed and reported to be downregulated in oral cancers (Table 3).

The pathways associated with these miRNAs were further analyzed using miRPath 4.0 to investigate the potential role of *LINC00944* in the miRNA sponge mechanism (Figure 8b). Overall, these findings suggest that *LINC00944* may play a role in targeting miRNAs associated with various cancer-related pathways, key signaling cascades, and mechanisms driving cancer progression.

Furthermore, we investigated whether the predicted candidate miRNAs, which are downregulated in oral cancer and are predicted to be targets of *LINC00944*, could regulate key mRNAs involved in the TNF-α/NF-κB signaling pathway. To address this, we analyzed the miRNA-mRNA interactions using four databases: miRwalk, miRDIP, MIRDB, and miRTarbase. We compiled a list of miRNAs and selected those that were identified as targets of key genes in at least three of the databases, which also appear in our candidate miRNAs list.

The prediction revealed that *NFKB1* interacted with hsa-miR-338-3p, hsa-miR-340-5p, and hsa-miR-9-5p, while *RELA* was targeted by hsa-miR-506-3p (Figure 8c and Figure S1). The interaction predictions indicated that *LINC00944*, *NFKB1*, and *RELA* possess binding sites for these miRNAs. Based on these observations, it can be suggested that *LINC00944* may regulate the TNF-α/NF-κB signaling pathway through competitively binding to target miRNAs that interact with *NFKB1* and *RELA*.

In our previous results, we observed that *LINC00944* promoted the conversion of M0 macrophages to M1 macrophage subtypes. By analyzing differentially expressed genes associated with M1 macrophage polarization from microarray data of SCC25-EBV cells, we identified that TNF-α and interferons (IFNs), known inducers of M1 polarization, were upregulated in EBV-positive SCC25 cells. Additionally, we identified increased mRNA expression of cytokines, chemokines, and transcription factors related to M1 polarization, including *IRF1*, *IRF5*, *IL-6*, *IL-1β*, *CCL3*, and *STAT1* (Figure 8d).

## 4. Discussion

EBV is a human tumor virus that has been linked to carcinogenesis through its latent proteins. The detection of EBV in OSCC tissues suggests that it may serve as a risk factor for oral cancer. However, the mechanisms by which EBV affects the development of OSCC are still debated and not fully understood. Recent studies in transcriptomics have emerged to help identify the mechanisms associated with carcinogenesis. LncRNAs exhibit abnormal expression in oral cancer and are correlated with disease progression. Additionally, alterations in lncRNAs can be influenced by viral proteins. Therefore, we proposed that EBV infection could potentially influence the initiation and progression of OSCC through its effects on cellular lncRNAs. Investigating the oncogenic roles of EBV-induced lncRNAs could offer us a better understanding of how EBV contributes to the development of oral cancer.

In this study, we focused on *LINC00944*, which has been linked to cancer progression and the presence of immune infiltrating cells in several types of cancer, including RCC [26,28], breast cancer [27], lung cancer [30], and gastric cancer [29]. However, the role of *LINC00944* in OSCC has not been previously investigated. To explore the association and function of *LINC00944* in oral carcinogenesis, we first examined its expression levels in HNSCC tissues, using data from the ImmREG database. Our observations revealed a significantly higher expression of *LINC00944* in HNSCC tissues compared to normal tissues. This finding suggested that *LINC00944* may play a role in the carcinogenesis of HNSCC. Since OSCC is a subtype of HNSCC, it is likely that *LINC00944* also influences OSCC. To further investigate the association of *LINC00944* with EBV infection, we analyzed its expression levels in oral cancer cell lines with and without EBV infection. We utilized two different clones of SCC25 cell lines, which serve as models for EBV infection. Our results showed that *LINC00944* was significantly high expressed in the EBV-positive oral cancer cell lines compared to the EBV-negative cell lines. This suggested that EBV infection may impact the expression of *LINC00944* in oral cancer cells. Taken together, these findings indicate that EBV may induce the expression of *LINC00944*, thereby influencing oral carcinogenesis.

Furthermore, the potential oncogenic roles of *LINC00944* were studied. In this study, we overexpressed *LINC00944* in oral squamous cell carcinoma cell lines including SCC25 and ORL-48T cells. To assess the migration ability of the cancer cells, we conducted a wound healing assay, while a transwell assay was utilized to evaluate their invasion capability. Our findings indicated that the overexpression of *LINC00944* promotes metastasis in oral cancer cells by enhancing both migration and invasion abilities. It is crucial to understand that lncRNA expression is tissue-specific [79], and a single lncRNA can have different functions depending on the pathways it targets in various cell types [18]. Previous studies of *LINC00944* functions showed that the increasing level of *LINC00944* promotes cancer progression in RCC [26], while a lower level of it correlates with the increasing tumor size [27]. In our study, we found that elevated levels of *LINC00944* promote the progression of oral cancer by increasing metastatic abilities. These observations align with the findings of Heawchaiyaphum, et al. (2020), which indicated that EBV-infected HSC1 and SCC25 cells enhance the metastasis of cancer cells [12]. It can be suggested that EBV may regulate the migration and invasion of oral cancer cells by influencing *LINC0994*, thereby promoting cancer progression.

The TME is vital in the initiation and progression of OSCC. The communication within the TME leads to immune evasion, angiogenesis, and treatment resistance, which consequently increases the advancement of cancers [32]. Based on this understanding, we hypothesized that EBV-induced *LINC00944* may facilitate cell-to-cell communication with neighboring cells through secretion, thereby promoting cancer progression. Our investigation revealed that *LINC00944* can be secreted from oral cancer cells that overexpress *LINC00944*. Incubating the culture medium containing secreted *LINC00944* with SCC25, ORL-48T, and THP-1 cells demonstrated that these recipient cells can internalize the secreted *LINC00944*. Moreover, the levels of *LINC00944* increased following this incubation. Taken together, these findings suggest that the effects of *LINC00944* appear to enhance its function not only in the originating cells but also in the surrounding neighboring cells. This could potentially lead to an increase in tumor progression and influence immune cells within the environment.

The TME consists of various components, including immune cells, CAFs, and endothelial cells. To investigate the influence of *LINC00944* on immune cells within the tumor microenvironment. The analysis of gene expression data for immune cells by ImmREG to examine the association of *LINC000944* with different subtypes of immune cells in HNSCC cases showed that the expression of *LINC00944* is associated with several immune cell types, including macrophages and M1 macrophages. In this study, we were interested in TAMs, which have been shown to play a significant role in OSCC and are also associated with EBV infection [33,34,42]. Regarding the results that the expression of *LINC00944* was upregulated in THP-1 cells after incubation with cultured medium containing secreted *LINC00944*, we observed an increase in M1 macrophage subtype markers compared to the control group. However, the observed up-regulation of *CD163*, an M2 marker, highlights the complexity of the response. It is important to note that a cancer cell-conditioned medium likely contains a mixture of biomolecules beyond *LINC00944*. Additionally, *LINC00944* showed high expression levels in M1 macrophages. Therefore, the direct role of *LINC00944* in macrophage differentiation was conducted. The directed effects of *LINC00944* were confirmed, indicating a change in macrophage subtype markers toward M1. Taken together, *LINC00944* induces the polarization of macrophages into the M1 subtype.

In the TME, TAMs such as M1 macrophages are known as anti-tumor subtypes, whereas TAMs like M2 macrophages are associated with promoting tumor growth. However, some studies have reported that M1 macrophages may also play a role in promoting carcinogenesis. In hepatocellular carcinoma (HCC), M1 macrophage infiltration in HCC tissues is linked to PD-L1 expression in HCC cells, induced by the inflammatory cytokine IL-1β secreted by M1 macrophages [37]. The studies of the association of M1-like TAMs and OSCC have been demonstrated. ASCT2 deletion in oral epithelium induces oxidative stress, faster OPMD to OSCC progression, and increased thrombospondin-1 (THBS1) expression, promoting M1-like TAM polarization via exosomes [80]. To engage more information in this, other studies demonstrated that OSCC-derived exosomes carrying THBS1 help polarize macrophages to an M1-like state, enhancing OSCC cell migration [40]. These M1-like TAMs secrete IL6, supporting EMT and cancer stem cells via the JAK/STAT3 pathway, thus creating a positive feedback loop [41]. Additionally, M1 macrophage-conditioned medium activates ErbB2/PI3K/AKT and MAPK/ErK pathways, with GDF15 mediating ErbB2 activation [39]. These findings revealed the new insight of M1-like TAMs in the role of OSCC progression promoter by activating an IL6/Stat3/THBS1 feedback loop, leading to a mesenchymal/stem-like phenotype [38].

While EBV may have several roles in oral cancers, we focused here on its impact on host cellular lncRNA. An integrated bioinformatics analysis of EBV-associated cell lines, including nasopharyngeal carcinoma, EBV-associated gastric carcinoma, and oral squamous cell carcinoma, identified the TNF-α/NF-κB signaling pathway as common hallmarks in EBV-associated epithelial cancers, providing the critical molecular insights into the mechanisms driving EBV-related epithelial malignancies [13]. Furthermore, the TNF-α/NF-κB signaling pathway has been shown to play a role in promoting the migration and invasion of OSCC [70]. Thus, we focus on the TNF-α/NF-κB signaling pathway. LncRNAs, including *LINC00944*, can function as miRNA sponges by binding to miRNAs that would typically target certain mRNAs for degradation. This mechanism is known as ceRNA, and it was a key mechanistic focus of our investigation. Therefore, the *LINC00944* interaction with miRNA, which specifically targets the NF-κB signaling pathway, was investigated and the M1 polarization-associated genes in SCC25-EBV cells from microarray were identified. From these findings, it can be suggested that EBV infects epithelial cells through specific glycoprotein interactions and modifies the expression of host genes, particularly those involving the TNF/NF-κB signaling pathway. The upregulation of certain lncRNAs is linked to miRNAs that target *NFKB1* and *RE*LA, resulting in increased levels of these genes. This leads to enhanced NF-κB/*RELA* complex formation, promoting transcription of pro-inflammatory genes associated with cancer progression, immune cell regulation, and macrophage polarization toward the M1 subtype. Additionally, cancer cells influenced by EBV can secrete various substances that contribute to their survival and alter the tumor microenvironment. Overall, EBV infection disrupts normal signaling pathways, fostering a supportive environment for cancer development.

The increase in NF-κB signaling pathway activation leads to the enhancement of cancer-associated gene transcription as well. In the present study, we found that *TNF*, *IFN*, *IRF1* and *IRF5*, *IL-6*, *IL-1β*, and *CCL3* were upregulated in EBV-positive SCC25 cancer cells compared to EBV-negative SCC25 cancer cells. These genes are associated with the promotion of cancer cell migration and invasion. The metastasis of tumors is facilitated by TNF-α, which exerts its influence on both the tumor cells themselves and the surrounding stromal and inflammatory cells within the tumor environment [81]. Moreover, TNF-α enhances the invasive characteristics of cancer cells through the induction of EMT, which is mediated by mechanisms dependent on Snail or ZEB1/ZEB2 [82,83,84]. The promotion of proliferation, oncogenic cytokine secretion, and aggressiveness of OSCC are associated with IL-1β. IL-1β also stimulated the secretion of oncogenic cytokines like IL-6, promoting angiogenesis [85]. Elevated levels of IL-6 were observed in OSCC tissue and associated with inflammatory cytokines, which led to the enhancement of OSCC progression through the JAK2/STAT3/Sox4/NLRP3 signaling pathway [86]. IL-6, in the tumor microenvironment of HNSCC, plays a role in mediating EMT and increasing the metastatic potential of tumor cells via the signaling pathway of JAK/STAT3/Snail [87]. Through IL-6R-mediated pathways, IL-6 has the potential to promote angiogenesis as well as tumor growth and progression [88]. CCL3 derived from esophageal squamous cell carcinoma (ESCC) cells and TAMs binds to CCR5, activating Akt and ERK pathways to promote ESCC cell invasion and migration [89]. Secreted CCL3 from hepatocytes, enhances cancer spread by triggering VIRMA and its key downstream target SIRT1, which drives the metastasis of intrahepatic cholangiocarcinoma [90]. In colon adenocarcinoma, IRF1 correlated with metastasis and the extent of immune cell infiltration, including CD8+ T cells, dendritic cells, T-helper 1 cells, and T cell exhaustion [91].

Moreover, the association of these genes and the induction of M1 macrophages also has been reported. M1 macrophages could be induced by multiple molecules, such as T helper (Th) 1 cytokine, tumor necrosis factor alpha (TNF-α), interferon gamma (IFN-γ), and lipopolysaccharide (LPS) [92]. The transcription factor IRF5, a marker for inflammatory macrophages, plays a critical role in macrophage polarization toward the pro-inflammatory M1 phenotype. In the antigen-induced arthritis mouse model, increased IRF5 expression was detected in macrophages at the site of inflammation, correlating with higher levels of pro-inflammatory markers, providing insights into the role of IRF5 in macrophage polarization and inflammation [93]. *IRF5* expression is induced by inflammatory stimuli like GM-CSF and IFN-γ and drives the production of M1-associated cytokines like IL-12, IL-23, and TNF while suppressing the anti-inflammatory cytokine IL-10. Macrophages expressing IRF5 promote the activation and proliferation of Th1 and Th17 cells, setting up a potent pro-inflammatory environment [94]. The study of IRF1 and IFN-β roles in M1 polarization of macrophages and their anti-tumor functions demonstrated that these two factors regulate IRF5 expression, contributing to M1 polarization. This indicates that the interplay among IRF1, IFN-β, and IRF5 is crucial for the M1 polarization of macrophages and their anti-tumor activities, providing potential targets for cancer therapy [95].

The IFNs also play roles in M1 macrophage polarization. Generally, there are three types of IFNs, types I and II are associated with immune cells while type III IFNs are associated with epithelial cells [96]. According to the DEGs from microarray analysis, Type III interferons or interferon lambda (IFN-λs) are a family of cytokines that induce an antiviral state in cells and were upregulated in EBV-positive SCC25 compared to EBV-negative SCC25. The study showed that macrophages are highly responsive to IFN-λ through the induction of the IFN-λ receptor IFNLR1 upon differentiation. Upon IFN-λ stimulation, macrophages adopt a pro-inflammatory phenotype, expressing various interferon-stimulated genes, cytokines, and chemokines. This enhances macrophage cytotoxicity and phagocytosis, while also recruiting and activating unresponsive lymphocytes like NK and T cells. IFN-λ promotes a Th1 chemokine profile in macrophages, facilitating NK and T cell chemotaxis and NK cell cytotoxicity [97]. Type I and Type III IFNs activate similar downstream pathways involving the JAK-STAT cascade, leading to the induction of IFN-stimulated genes [98]. The STAT family includes a key transcription factor that regulates macrophage polarization. Upon activation of the IFN receptor, IFN-γ binding induces Janus kinase 1/2-mediated phosphorylation, leading to the activation of STAT1, which then binds to M1 promoters [99,100,101,102]. Research has demonstrated that IL-6 can stimulate both M1 and M2 pathways [103,104,105]. However, other factors, including clinical staging as well as the difference of IL-6, also influence macrophage polarization [106].

In the study of the role of macrophage polarization in the increased radiosensitivity of human papillomavirus (HPV)-positive HNSC. HPV-positive HNSC cells promote M1 macrophage polarization and secrete higher levels of the cytokine IL-6, further enhancing this polarization [107]. Chemokines play a crucial role in regulating macrophage differentiation and polarization. In the tumor microenvironment, chemokines such as CCL2, CCL3, CCL4, and CCL5 attract monocytes that differentiate into TAMs [108]. That study examined the role of the chemokine CCL3 in nonalcoholic fatty liver disease, finding that it colocalizes with M1-like pro-inflammatory macrophages in the liver, indicating its involvement in macrophage infiltration and polarization [109].

Our findings highlight the crucial role of EBV-induced *LINC00944* in the progression of oral cancer. This factor not only promotes the migration and invasion of oral cancer cells directly but also influences the surrounding TME by polarizing macrophages into M1-like TAMs. These findings point to a new target for studying its function in cancer and could provide valuable insights for therapeutic research. Recently, several lncRNAs have emerged as promising targets for treatment. Suppressing lncRNAs such as *NEAT1*, *MALAT1*, and *UCA1* through various methods including CRISPR/Cas9, siRNA, or antisense oligonucleotides has been shown to impede cancer development [110]. Moreover, ongoing clinical trials explore the potential of *EGFR-AS1* and its target EGFR gene, as well as *MALAT1* and its target miR-124, as biomarkers and innovative therapeutic targets in oral cancer [111]. Consequently, further exploration of *LINC00944*’s mechanism in oral carcinogenesis is essential, as a deeper understanding could lead to the identification of additional potential biomarkers and therapeutic targets.

**Figure 9:**
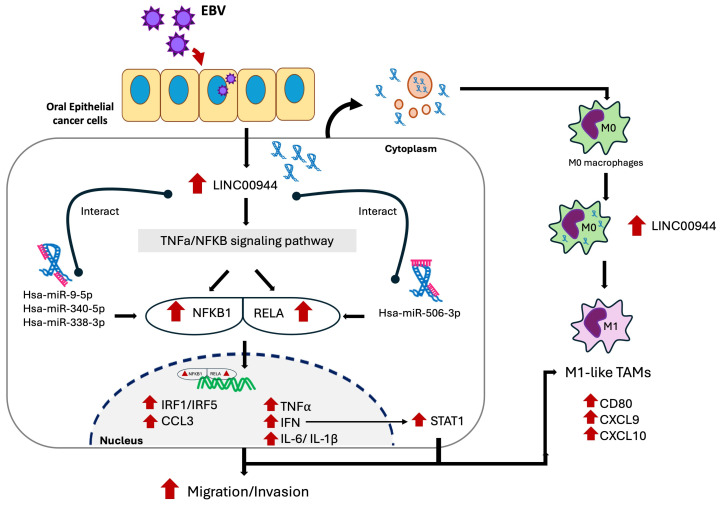
The proposed mechanism elucidating the involvement of EBV in oral cancers encompasses the regulation of host cellular lncRNA *LINC00944*. EBV infection modulates the expression of host genes, including up-regulation of *LINC00944*, which is associated with TNF/NF-κB signaling pathway. The *LINC00944* interacts with miRNAs targeting *NFKB1* (hsa-miR-340-5p, hsa-miR-9-5p, and hsa-miR-338-3p) and *RELA* (hsa-miR-506-3p) in a competitive manner, inhibiting their regulation. This leads to elevated levels of *NFKB1* and *RELA*, promoting the formation of the NF-kB/*RELA* complex, which translocates to the nucleus and increases transcription of downstream genes (*TNF, IFN, IRF1, IRF5, IL-6, IL-1β, CCL3*) associated with inflammation and cancer progression. These targets also activate STAT1, enhancing macrophage polarization toward the M1 subtype and further supporting cancer progression. Additionally, *LINC00944* is secreted by cancer cells, influencing neighboring cells and immune cells within the TME, promoting macrophage polarization to M1, and contributing to the cancer-promoting microenvironment. In summary, EBV infection increases *LINC00944*, affecting TNF-α/NF-κB signaling via the lncRNA-miRNA-mRNA axis, driving cancer progression and immune cell regulation.

Overall, to summarize our findings, the proposed mechanisms by which EBV contributes to oral cancer involve the regulation of host cellular lncRNA, as demonstrated by *LINC00944* (Figure 9).

These findings provided valuable new insights into the role of *LINC00944* in EBV infection and OSCC migration and invasion. Its association with macrophage polarization and the proposed mechanism was demonstrated. However, the proposed mechanism involving *LINC00944*-miRNA-*NFKB1/RELA* interactions is based on bioinformatic predictions. Therefore, experimental validation of these interactions through in vitro studies is necessary to confirm their functional significance in OSCC progression. Such validation would strengthen the findings and contribute to a deeper understanding of the underlying mechanisms, which would be beneficial for studying OSCC development and progression, as well as for therapeutic interventions.

## 5. Conclusions

This study examined the role of *LINC00944* in OSCC and its association with cancer progression. Upregulation of *LINC00944* in EBV-associated OSCC contributes to oral cancer progression by promoting migration and invasion through one of its functions as ceRNA, which targets specific miRNAs that regulate proteins in the TNFα/NF-κB signaling pathway. This results in increased *NFKB1* and *RELA* expression and may contribute to the upregulation of M1 markers in THP-1 cells, indicating polarization toward the M1 macrophage subtype in the tumor microenvironment. However, it is important to note that the proposed mechanisms are based on evidence from our study, previous research data, and predictive tools. In vitro studies on lncRNA-miRNA-mRNA interactions are needed to strengthen these findings. Overall, our insights deepen the understanding of EBV’s role in oral cancer and may aid in identifying new treatment targets.

## Figures and Tables

**Table 1:** Primer sequences.

Gene Name	Forward (5′-3′)	Reverse (5′-3′)
*LINC00944*	ATGCTGGAAGAACGAGCCTT	GGCCCTGGTCCTAGGTCATA
*CD80*	ATCCTGGGCCATTACCTTAATC	CTCTCATTCCTCCTTCTCTCTCT
*CXCL9*	GCTGGTTCTGATTGGAGTGC	GAAGGGCTTGGGGCAAATTG
*CXCL10*	CCTTATCTTTCTGACTCTAAGTGG	CTAAAGACCTTGGATTAACAGG
*CD206*	GTTACCCTGGTGGAAGAAGAAG	CTCGTTTACTGTCGCAGGTATC
*CD163*	TTTGTCAACTTGAGTCCCTTCAC	TCCCGCTACACTTGTTTTCAC
*ALOX15*	CAGATGTCCATCACTTGGCAG	CTCCTCCCTGAACTTCTTCAG
*GAPDH*	TCATCAGCAATGCCTCCTGCA	TGGGTAGCAGTGATGGCA

**Table 2:** *LINC00944*-associated immune cells in HNSCC.

Immune Cell	Analysis Tools	*p*-Value
Plasmacytoid dendritic cell	XCELL	7.32 × 10^−27^
T cell CD8+	TIMER	7.26 × 10^−25^
	MCPCOUNTER	7.20 × 10^−20^
	QUANTISEQ	1.40 × 10^−19^
	XCELL	2.28 × 10^−17^
	CIBERSORT	2.11 × 10^−15^
NK cell	MCPCOUNTER	2.32 × 10^−18^
	EPIC	8.25 × 10^−16^
Myeloid dendritic cell	TIMER	1.10 × 10^−17^
Macrophage M1	CIBERSORT	4.61 × 10^−17^
	QUANTISEQ	4.49 × 10^−13^
NK cell activated	CIBERSORT	5.05 × 10^−16^
Myeloid dendritic cell activated	XCELL	5.59 × 10^−16^
Immune score	XCELL	5.55 × 10^−15^
T cell CD4+ Th1	XCELL	1.55 × 10^−14^
T cell CD8+ effector memory	XCELL	2.28 × 10^−14^
Cytotoxicity score	MCPCOUNTER	3.77 × 10^−14^
Class-switched memory B cell	XCEL	1.84 × 10^−13^
Macrophage	EPIC	3.43 × 10^−13^
T cell CD4+ (non-regulatory)	QUANTISEQ	4.49 × 10^−13^

**Table 3:** Downregulated miRNAs in oral cancer targeted by *LINC00944* through interaction prediction.

miRNAs	Cells	Samples	Functions	References
hsa-miR-26a/b	SAS and HSC3 cell lines	36 pairs of matched primary OSCC and normal epithelial tissue	Loss of tumor-suppressive miR-26a/b enhanced cancer cell migration and invasion in OSCC through direct regulation of *TMEM184B*.	[71]
hsa-27a-3p	Tca8113, CAL-27, SCC-4, SCC-9, SCC-25, HN-6 compared to HNOK cell lines	50 pairs of matched primary OSCC and adjacent non-cancerous tissue	miR-27a-3p acts as an important upstream regulator related to the EMT via the inhibition of *YAP1* in OSCC	[72]
hsa-miR-125b	Ten OSCC cell lines compared to NHOK controls	9 of OSCC tumors compared and 5 of NHOK controls	Transfecting cells with exogenous miR-125b and miR-100 significantly reduced cell proliferation and modified the expression of target and non-target genes, including some that are overexpressed in radioresistant OSCC cells	[73]
hsa-miR-1271	SCC4 and Tca8113 cells compared to normal oral mucosa cells	20 pairs of matched primary OSCC and normal epithelial tissue	Overexpression of miR-1271 inhibited cell proliferation, migration, and invasion in OSCC. miR-1271 inhibits OSCC cell growth and metastasis by targeting *ALK*	[74]
hsa-miR-338	Tca-8113 cells and SCC15 cells compared to normal oral epithelial cells	24 pairs of matched primary OSCC and normal epithelial tissue	Overexpression of miR-338 significantly inhibited proliferation, colony formation, migration, and invasion of OSCC cells via targeting *NRP1*	[75]
hsa-miR-340	SAS and SCC15 compared to oral epithelial cells	3 pairs of matched primary OSCC and normal adjacent tissues	Decrease in miR-340 increased *Glut1* expression, leading to an increase in lactate secretion and glucose uptake rate which resulted in the rapid proliferation of oral cancer cells.	[76]
hsa-miR-9	-	Serum from 104 of OSCC patients or 30 of OLK in comparison with 40 of healthy controls	Low serum miR-9 was correlated with poor prognosis of OSCC	[77]
hsa-miR-506	SCC4 and Tca8113 cells compared to normal oral mucosa cells	21 pairs of matched OSCC and adjacent normal tissues	miR-506 overexpression suppressed proliferation, migration, and invasion capabilities of OSCC cells via targeting *GATA6*	[78]

## Data Availability

The microarray datasets used in the current study are available from the corresponding author upon reasonable request.

## Supplementary Materials

The following supporting information can be downloaded at: https://www.mdpi.com/article/10.3390/cancers17030491/s1, Spreadsheet xlsx file Table S1: Microarray_Genes in TNFa-NFkb pathway_Normalized data and Figure S1: The predicted binding site between miRNA and target.
