# Supplementary material for: EBV-Induced LINC00944: A Driver of Oral Cancer Progression and Influencer of Macrophage Differentiation
Source: Cancers (Basel). 2025 Feb 1;17(3):491. doi: 10.3390/cancers17030491 (PMC11815735; doi:10.3390/cancers17030491)
Supplement: Supplementary file 1 [file cancers-17-00491-s001.zip › Supplementary Figure.pdf]

Supplementary Figure

(a)

```
Target (top)     : LINC00944
Query  (bottom)  : hsa-miR-9-5p

            552       560
              |         |
5'-CUU..CCCUU           UAUGU..AGG-3'
              UAUAGCUGG
              |||||||||
              AUGUCGAUC
        3'-AGU          UAUUG..UCU-5'
              |         |
             20         12
```

(b)

```
Target (top)     : LINC00944
Query  (bottom)  : hsa-miR-340-5p

          214               227
            |                 |
5'-CUU..GAAAC             U   CUAUA..AGG-3'
             AUCA UCUCAUU GC
             |||| ||||||| ||
             UAGU AGAGUAA CG
       3'-U      C            AAAUAUU-5'
            |                 |
           21                 8

          540                555
            |                  |
5'-CUU..CACCU    GGCCC         GCUGG..AGG-3'
             UCAU      CUUUAUA
             ||||      |||||||
             AGUA      GAAAUAU
  3'-UUAGUCAG    AC            U-5'
            |                  |
           14                  2
```

(c)

```
Target (top)     : LINC00944
Query  (bottom)  : hsa-miR-338-3p

           257      264
             |        |
5'-CUU..UAGAA         AGAAC..AGG-3'
             AUGCUGGA
             ||||||||
             UACGACCU
3'-GUU..GUGAC
             |        |
             8        1
```

(d)

```
Target (top)     : LINC00944
Query  (bottom)  : hsa-miR-506-3p

           111      117
             |        |
5'-CUU..UUCGG         AAGAC..AGG-3'
             UCGGAAG
             |||||||
             AGUCUUC
     3'-AGAUG         CCACGGAAU-5'
             |        |
            16        10
```

(e)

```
Target (top)     : NFKB1
Query  (bottom)  : hsa-miR-9-5p

          699      705
            |        |
5'-CCU..AAAUU        UCAAA..AAA-3'
             GAUGACC
             |||||||
             CUAUUGG
3'-AGU..UCGAU        UUUCU-5'
            |        |
           12        6
```

(f)

```
Target (top)     : NFKB1
Query  (bottom)  : hsa-miR-340-5p

          294               308
            |                 |
5'-CCU..UGCUU  U  UG          GUUGU..AAA-3'
             UC GU  UCAUUGCU
             || ||  ||||||||
             AG CA  AGUAACGA
      3'-UU  U  G             AAUAUU-5'
            |                 |
           20                 7

          554      561
            |        |
5'-CCU..UACAA        GAAAA..AAA-3'
             UCAUUGCU
             ||||||||
             AGUAACGA
  3'-UUAGUCAG        AAUAUU-5'
            |        |
           14        7

          662               680
            |                 |
5'-CCU..CUAUU        UUA  U   UAAAA..AAA-3'
             UUUUAUU    CU UUAUAA
             |||||||    || ||||||
             AGAGUAA    GA AAUAUU
    3'-UUAGUC          C
            |                 |
           16                 1
```

(g)

```
Target (top)     : NFKB1
Query  (bottom)  : hsa-miR-338-3p

          432            445
            |              |
5'-CCU..AAAUG          U   UGCCA..AAA-3'
             AGAGUCACU GAUG
             ||||||||| ||||
             UUUUAGUGA CUAC
     3'-GUUG               GACCU-5'
            |              |
           18              6

          639      646
            |        |
5'-CCU..AAAAA        UUUAU..AAA-3'
             AUGUUGGA
             ||||||||
             UACGACCU
3'-GUU..GUGAC
            |        |
            8        1
```

(h)

```
Target (top)     : RELA
Query  (bottom)  : hsa-miR-506-3p

          622                 638
            |                   |
5'-GGG..CUCCC   U    A          AUAGU..AAA-3'
             UCU CUCA     GUGCCUUA
             ||| ||||     ||||||||
             AGA GAGU     CACGGAAU
        3'-     U    CUUCC
            |                   |
           21                   1

          691      698
            |        |
5'-GGG..UCCAG        CAUAG..AAA-3'
             UCAGGAGG
             ||||||||
             AGUCUUCC
     3'-AGAUG        CACGGAAU-5'
            |        |
           16        9

          725      731
            |        |
5'-GGG..AAAGC        CUCUU..AAA-3'
             ACUUGGA
             |||||||
             UGAGUCU
       3'-AGA        UCCCA..AAU-5'
            |        |
           18        12
```

Figure S1. The predicted binding site between miRNA and target. Interaction between *LINC00944* and (a) hsa-miR-9-5p, (b) hsa-miR-340-5p, (c) hsa-miR-338-3p, and (d) hsa-miR-506-3p. Interaction between *NFKB1* and (e) hsa-miR-9-5p, (f) hsa-miR-340-5p, and (g) hsa-miR-338-3p. Interaction between *RELA* and (h) hsa-miR-506-3p.
